# Anti-cancer Potential of Polysaccharide Extracted From *Polygonatum sibiricum* on HepG2 Cells via Cell Cycle Arrest and Apoptosis

**DOI:** 10.3389/fnut.2022.938290

**Published:** 2022-07-04

**Authors:** Mo Li, Yumeng Liu, Henan Zhang, Yanfeng Liu, Weiming Wang, Shengbo You, Xinyu Hu, Meijun Song, Rina Wu, Junrui Wu

**Affiliations:** ^1^College of Food Science, Shenyang Agricultural University, Liaoning Engineering Research Center of Food Fermentation Technology, Shenyang Key Laboratory of Microbial Fermentation Technology Innovation, Shenyang, China; ^2^College of Criminal Science and Technology, Criminal Investigation Police University of China, Shenyang, China; ^3^Heilongjiang Academy of Traditional Chinese Medicine, Harbin, China; ^4^Institute of Crop Germplasm Resources, Shandong Academy of Agricultural Sciences, Jinan, China

**Keywords:** *Polygonatum sibiricum* polysaccharide, structural characterization, antitumor activity, HepG2 cells, cell apoptosis

## Abstract

*Polygonatum sibiricum* is one of the most widely used traditional Chinese medicine in China. *Polygonatum sibiricum* polysaccharide (PSP) is the main functional component of *Polygonatum sibiricum*. In this study, a water-soluble polysaccharide (PSP-1) was first isolated from *Polygonatum sibiricum* with a molecular weight of 38.65 kDa. Structural analysis was performed via methylation and FT-IR spectroscopy analyses, which in combination with NMR spectroscopy, revealed that PSP-1 has a → 4-α-D-Glcp-1 → backbone with the substitution at O-6 with the β-D-Glcp-1 → residues. Furthermore, PSP-1 exhibited potent and concentration-dependent anticancer effects, inducing HepG2 cell apoptosis and arresting the cell cycle at the G1 phase. Moreover, PSP-1 also decreased the mitochondrial membrane potential, damaged the nucleus of HepG2 cells, and increased the activity of caspase-9 and−3 in the intrinsic apoptotic pathways to induce HepG2 cell apoptosis. To conclude, PSP-1 might be a good candidate for the treatment of liver cancer, and this work provides important information for understanding the relationship between structure and antitumor activity of PSP-1, which is relevant for the treatment of hepatocellular carcinoma in clinic.

## Introduction

Hepatocellular carcinoma (HCC) is a leading cause of cancer-related mortality worldwide. Traditional methods for the treatment of HCC consist of surgery and medication. However, over 80% of HCC patients are not suited to surgical treatment due to severe liver injury (1). Although medication has an initial therapeutic effect, the long-term use of anti-cancer drugs commonly gives rise to drug resistance, in addition to being associated with various side effects (2). Therefore, the development of highly effective and non-toxic natural compounds is seen as a good alternative for the treatment of liver cancer. In recent years, many studies have shown that natural polysaccharides have significant inhibitory effects on tumors (3–6). Natural polysaccharides have become the main research focus for anti-tumor drugs since they are less toxic and have no side effects in humans.

*Polygonatum sibiricum* (PS) extracts are associated with many beneficial pharmacological functions and are used widely in treatments against hypercholesterolemia, diabetes, cancer, and inflammatory diseases, among others (7, 8). Of these, polysaccharides play a significant role in PS-based therapeutics (9). The biological activities of polysaccharides are closely related to their complex structure, including their molecular weight, monosaccharide composition, glycosidic linkages, substituents, and degree of branching (10–12). For instance, the high-molecular-weight (>300 kDa) polysaccharides of *Hirsutella sinensis* and *Ganoderma lucidum* are known to play a predominant against obesity (13, 14), while the low-molecular-weight polysaccharides of *Tremella fuciformis* and *Enteromorpha linza* have been shown to exhibit high levels of antioxidant activity (15, 16). Furthermore, polysaccharides with a high uronic acid content have been shown to exert significantly high levels of antioxidant activity (17). Therefore, the identification of the polysaccharide structure is essential to determine the relationship between the structure and biological activities of these molecules.

In few studies, it was revealed that *Polygonatum sibiricum* polysaccharide (PSP) have inhibitory effects on lung cancer and cervical cancer (8, 18). In addition, PSP also has protective effect on liver, which can significantly alleviate the chemical damage of hard storage and prevent the acute heart failure induced by adriamycin which indicate that PSP can act on the liver to resist some diseases (19, 20). Nevertheless, less is known for the anticancerous effects of PSP against HCC and the relationship between structure and antitumor activity. Therefore, there is an urgent need to characterize the structure and antitumor activity of PSP.

In the present study, a water-soluble polysaccharide (PSP-1) was extracted and purified from *Polygonatum sibiricum* using Sephrose DEAE-52 cellulose and Sephadex G-100 HR column chromatography. The structure of PSP-1 was analyzed using High performance liquid chromatography (HPLC), Gas Chromatography and Mass Spectrometry (GC-MS), Fourier-transform infrared spectroscopy (FT-IR), Scanning electron microscopy (SEM), and Nuclear magnetic resonance (NMR) spectroscopy. Furthermore, *in vitro* experiments using human hepatoma HepG2 cells were conducted to assess the effect of PSP-1 on the proliferation, metastasis, and apoptosis of HepG2 cells.

## Materials and Methods

### Materials

*Polygonatum sibiricum* were purchased from a local market (Shenyang, Liaoning, China). HepG2 cell line was purchased from Stem Cell Bank, Chinese Academy of Sciences. DEAE-52 cellulose and Sephadex G-100 were obtained from Solarbio Co., Ltd (Beijing China). Monosaccharide standards (D-glucose, D-mannose, D-galactose, L-arabinose, D-xylose, L-rhamnose, and D-fucose) were purchased from Shanghai yuanye Bio-Technology Co., Ltd (Shanghai China). Fetal bovine serum (FBS), Dulbecco's Modified Eagle Medium (DMEM), trypsin EDTA, penicillin and streptomycin were obtained from Gibco (Grand Island, NY, USA). Caspase-3 and caspase-9 kit were purchased from KeyGEN Biotechnology Co., Ltd. (Jiangsu, China). All other chemicals were of reagent grade.

### Extraction and Purification of PSP

*Polygonatum sibiricum* was washed and dried at 50°C for 48 h. The dried *Polygonatum sibiricum* was then crushed into a powder using a micronizer and filtered through a 100-mesh sieve. The resulting powder was pretreated with petroleum ether at 25°C for 6 h to remove any fats, followed by extraction using 85% ethanol for 24 h to remove any pigments and small organic compounds. After suction filtration, the filter residue was dried at 50°C and extracted using distilled water (1:20, g/mL) at 90°C for 2 h under continuous stirring. The extraction solution was centrifuged (4,000 rpm, 15 min), and the resulting supernatant was concentrated to an appropriate volume by decompression at 70°C in a rotary evaporator. The supernatant was precipitated with ethanol (1:4, v/v) for 12 h, collected by centrifugation (4,000 rpm, 10 min), and deproteinized using Sevag reagent. The extracts were precipitated again using ethanol (1:4, v/v) for 12 h and centrifuged (4,000 rpm, 10 min). Lastly, the precipitates were dialyzed against water for 48 h (cut-off Mw 8–14 kDa) and lyophilized as crude polysaccharides.

**Figure 1:**
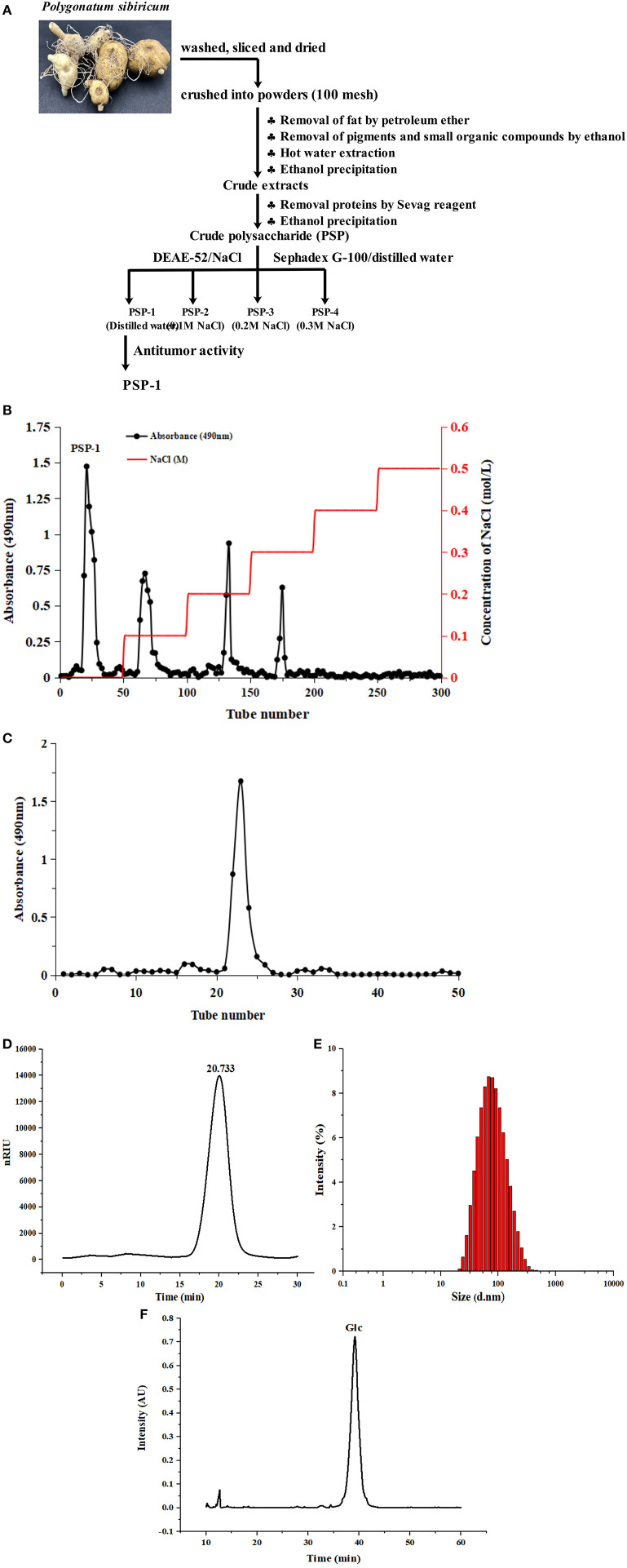
Diagram of extraction process of *Polygonatum sibiricum* polysaccharide fraction 1 (PSP-1) **(A)** Chromatography of PSP-1 by DEAE-52 cellulose column **(B)** and Sephadex G-100 chromatography **(C)** HPGPC chromatograms of PSP-1 **(D)** Particle size distribution of PSP-1 **(E)** Monosaccharide composition of PSP-1 **(F)**.

The crude polysaccharides were dissolved in distilled water and loaded onto a DEAE-52 cellulose column (2.6 ×40 cm), and eluted stepwise using NaCl solutions (0, 0.1, 0.2, 0.3, 0.4, and 0.5 M) at a flow rate of 1 mL/min. The sugar elute was collected in tubes (5 min/tube) and then combined under the monitor using the phenol sulfuric acid method. The major fractions were concentrated, dialyzed (cut-off Mw 3,500 Da), and lyophilized. The dried polysaccharides were dissolved for further purification using a Sephadex G-100 gel filtration column (1.6 ×30 cm) at a flow rate of 1 mL/min. The top fractions of the elution peak (5 min/tube) were collected and lyophilized to obtain the purified polysaccharides. The overall procedure used to purify PSP is illustrated schematically in Figure 1A. The extraction yield of the polysaccharides was calculated using the following formula:


(1) $$\begin{array}{r} \text{Extraction yield}\left(\text{\%}\right)\;=\;\left(W_{1}/W_{0}\right)\times 100 \end{array}$$

where W_1_ is the weight of dried crude polysaccharides and W_0_ is the weight of dry *Polygonatum sibiricum*.

The PSP-1 with the highest antitumor activity in different purified single-component polysaccharides which was selected by MTT assay (data not provided). In this study, we focused on the research of PSP-1.

### Characterization of PSP-1

#### Chemical Composition Analysis

Contents of total carbohydrates, uronic acid, and protein in PSP-1 were determined by the phenol-sulphuric acid assay (21), m-hydroxydiphenyl assay (22), and coomassie brilliant blue assay (23), respectively.

#### Monosaccharide Composition Analysis

The monosaccharide composition of PSP-1 was analyzed by high-performance liquid chromatography (HPLC), as described previously (24) and the detail methods were presented in Supplementary Method S1.

#### Molecular Weight Distribution and Particle Size Analysis

The molecular weight distribution of PSP-1 was determined by HPGPC as described before. The PSP-1 was dissolved in ultrapure water (4.0 mg/mL) and filtered through a 0.45 μm membrane before injection. Then 10 μL sample solution was analyzed by HPGPC-RID.

Particle size distributions of PSP-1 solution (1 mg/mL) were done using A Zetasizer Nano-ZS90 (Malvern, Britain) at 25°C.

#### Scanning Electron Microscopy (SEM) Analysis

The PSP-1 samples were thinly coated with gold powder and visualized by an SEM system (S4800, Tokyo, Japan).

#### Fourier Transform Infrared (FI-IR) Analysis

The infrared spectrum of PSP-1 was determined using a FT-IR (Nicolet 6700, Thermo Scientific, USA) within the frequency range of 4,000-400 cm^−1^.

#### Methylation Analysis

Methylation analysis of PSP-1 was performed according to a previously described method with some modifications (10) and the specific experimental methods were presented in Supplementary Method S2.

#### Nuclear Magnetic Resonance (NMR) Spectroscopy Analysis

The ^1^HNMR spectra and ^13^CNMR spectra of PSP-1 were recorded using a Bruker AV-400 NMR spectrometer (Bruker Instrumental Inc.,Billerica, Massachusetts, USA) at 25°C. Before measurement, the PSP-1 was dissolved with D_2_O.

### Antitumor Activity of PSP-1

#### Cell Culture

The HepG2 cells were cultured in DMEM with FBS (10% v/v) and penicillin (1% v/v) in 5% CO_2_ humidified atmosphere at 37°C.

#### Cell Proliferation Assay and Colony Formation Assay

The effect of PSP-1 on HepG2 cell proliferation was assessed using the MTT assay and the detail methods were presented in Supplementary Method S3.

#### Cell Migration Assay

The effect of PSP-1 on tumor cell migration was assessed using a wound-healing assay (25) and the detail methods were presented in Supplementary Method S4.

#### The Morphology of HepG2 After Treatment With PSP-1

Cell suspensions (1 mL) (5 × 10^5^ cells/mL) were inoculated in six-well-plates for 24 h. The culture medium was removed, and 1 mL PSP-1 solution (0, 100, 200, and 400 μg/mL) was added and incubated for 72 h. The supernatant was removed, washed with PBS three times, followed by the addition of acridine orange (AO) and ethidium bromide (EB) double staining kits for staining for 10–15 min. Observation was performed using confocal laser scanning microscopy (CLSM). AO passes through the cell membrane of living cells, chimeric with DNA, and shows green fluorescence under CLSM. EB cannot cross the cell membrane of living cells, but is able to cross the cell membrane of apoptotic cells and show red fluorescence.

The HepG2 cell suspension (1 mL) (5 × 10^5^ cells/mL) was inoculated in six-well-plates for 24 h. The culture medium was removed, and 1 mL of PSP-1 solution (0, 100, 200, and 400 μg/mL) was added and incubated for 72 h. HepG2 cells were then stained with Hoechst 33,342 (1 μg/mL) for 15 min at 37°C. After washing with PBS three times, nuclear morphology was observed under CLSM.

#### Mitochondrial Membrane Potential (MMP) Level

HepG2 cells were seeded in six-well-microplates and incubated at 37°C for 24 h to allow the cells to adhere. Then, PSP-1 was added and incubated for 72 h. The cells were harvested and washed with PBS, followed by incubation with JC-1 (1 mL) for 20 min at 37°C in the dark. The cells were covered with anti-fluorescence quenching agents after washing and observed by CLSM.

#### Cell Cycle and Apoptosis Assay

The detail methods of the cell cycle and apoptosis assay were presented in Supplementary Method S5

#### Determination of Apoptosis-Related Enzyme Activity

HepG2 cells were seeded in 6-well-plates. The PSP-1 solution (0, 100, 200, 400 μg/mL) was added. After incubation for 72 h, the cells were washed and lysed by lysis. The caspase-3 and caspase-9 activities were measured according to the guidelines for the caspase-3 and caspase-9 activity assay kits (C1116, C1158; Beyotime Biotechnology).

### Statistical Analysis

All experiments were conducted three times. The data were analyzed by SPSS 19.0 statistical software. The experimental results were expressed as mean ± standard deviation. The difference was *p* < 0.05.

## Results and Discussion

### Physicochemical Properties and Monosaccharide Composition of PSP-1

PSP-1 was isolated using a DEAE-52 cellulose column and Sephadex G-100 after elution with distilled water. The resulting elution profile is shown in Figures 1B,C. The yield of PSP-1 was 3.01 ± 0.29%, based on the dry matter, with a total carbohydrate, protein and uronic acid content of 93.63 ± 1.81%, 0.44 ± 0.06%, and 0.98 ± 0.21%, respectively. The molecular weight of PSP-1 was measured by HPGPC using dextran as a standard, as shown in Figure 1D, which indicated that PSP-1 was a homogeneous polysaccharide. Based on the calibration curve (y = −0.2959x + 10.722, *R*^2^ = 0.9926), the molecular weight of PSP-1 was determined to be 38.65 kDa. In a previous study, Zhang et al. isolated a polysaccharide PS-WNP from *Polygonatum sibiricum* and determined its molecular weight to be 76 kDa (15). Wang et al. reported that a novel polysaccharide (PSP3) with the molecular weight of 7.743 kDa was isolated and purified from *Polygonatum sibiricum* (7). Different molecular weight distributions indicate that there are significant differences among polysaccharides (24, 26). In the present study, the molecular weight of PSP-1 was found to differ from other reported polysaccharides from *Polygonatum sibiricum*. Several studies have shown that the molecular weight of polysaccharides is closely related to their antitumor activity (10, 27).

The size distribution of PSP-1 in aqueous solution (Figure 1E) revealed a symmetrical narrow peak at approximately 101.3 nm. The polydispersity index of PSP-1 (0.276), which measures the uniformity of the particle size distribution of a polymer, indicated that PSP-1 was a homogenous polysaccharide.

The monosaccharide composition of PSP-1 was analyzed using HPLC. As shown in Figure 1F, PSP-1 was composed of glucose.

### Structure of PSP-1

#### SEM Analysis of PSP-1

**Figure 2:**
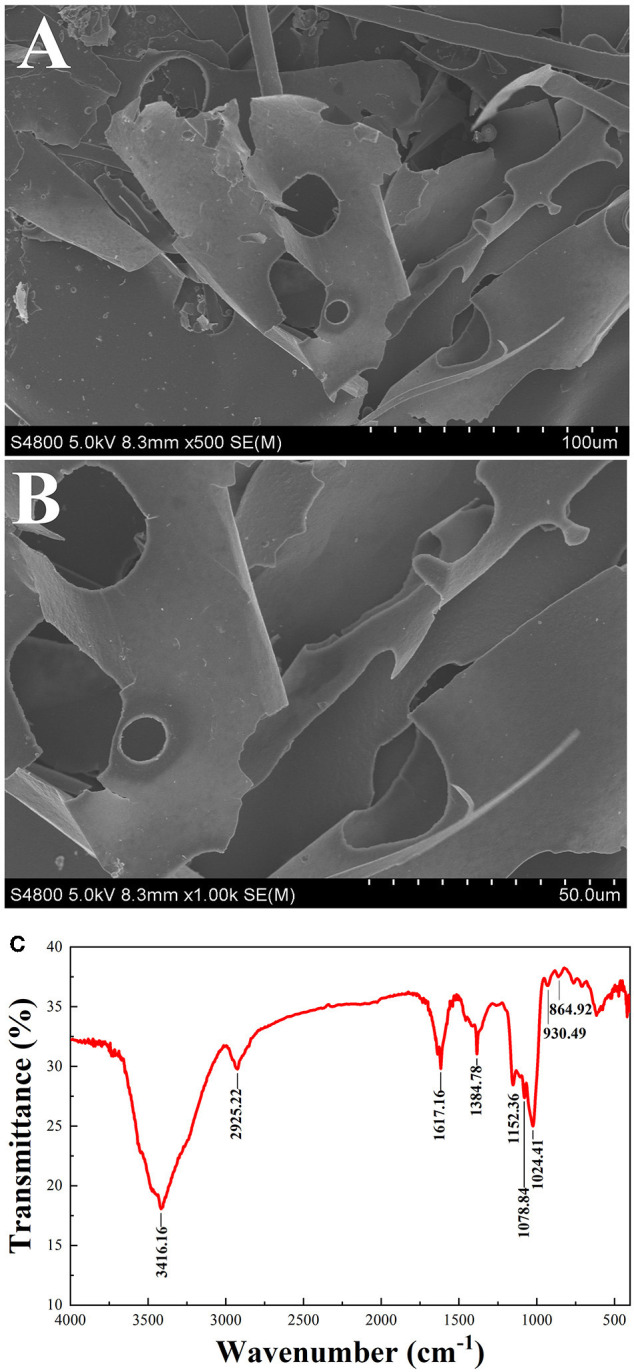
SEM (500 × 1,000 ×) of PSP-1 **(A,B)** FT-IR spectra of PSP-1 in the range of 4,000–400 cm^−1^ **(C)**.

The microstructure of PSP-1 was determined by SEM at a magnifications of 5.00 × and 10.00 ×, as shown in Figures 2A,B. PSP-1 exhibited a smooth and porous surfaces consisting of flake-like lamellae. Seen from the image at 1,000 × augmentation, a flake layer with unregular curls was observed in polygahatous polysaccharides. Representative Glc-rich natural polysaccharides show the aforementioned lamellar structure (28). The apparent structure of polysaccharides is closely related to the solubility and water absorption of polysaccharides (29). In addition, polysaccharides with an even and smooth sheet-like structure have better antitumor activity (25).

#### FT-IR Analysis

Infrared spectroscopy is commonly used to identify the primary structure of polysaccharides (30). The infrared absorption spectrum of PSP-1 is shown in Figure 2C. The sample was found to have a broad and strong absorption peak at 3416.16 cm^−1^, indicating that there is O-H group stretching vibration. The absorption band at 2925.22 cm^−1^ indicates that there is an asymmetric CH_2_ stretching vibration (31). The peaks were observed at 1617.16 cm^−1^ and 1384.78 cm^−1^ may be due to the symmetrical stretching vibration of COO- (32). The three absorption peaks observed at 1152.36, 1078.84, and 1024.41 cm^−1^ indicate the presence of pyran rings in the PSP-1. The characteristic absorption bands at 930.49 cm^−1^ and 864.92 cm^−1^ indicated that PSP-1 contained α- and β-anomeric units (24, 26, 33). These results confirmed that PSP-1 showed typical polysaccharide absorption peaks.

#### Methylation Analysis of PSP-1

PSP-1 was methylated, depolymerized, and analyzed by GC–MS to determine the linkage patterns of the sugar units (Table 1), indicating the presence of two components named 2,3,6-Me_3_-Glcp, 2,3,4,6-Me_3_-Glcp and 2,3-Me_2_-Glcp were shown in Table 1. These compounds are in a ratio of 79.18: 10.32: 10.50. Based on their comparison with mass spectrum patterns from the literature (34–36), and the standard data, the linkages of Glc were deduced to be → 4)-α-D-Glcp-(1 →, D-Glcp-(1 → and → 4,6)-α-D-Glcp-(1 →, respectively.

**Table 1:** Glycosyl linkages analysis of PSP-1.

**Numbers**	**Characteristic fragments (m/z)**	**Methylated sugar**	**Molar ratio (%)**	**Linkage type**	**References**
1	43, 45, 57, 85, 87, 99, 101, 113, 117, 161, 173, 233	2,3,6-Me_3_-Glcp	79.18	→ 4)-Glcp-(1 →	Gou et al. (34)
2	59, 71, 87, 102, 118, 129,145, 162,175, 205	2,3,4,6-Me_3_-Glcp	10.32	D-Glcp-(1 →	Zeng et al. (35)
3	57, 85, 99, 101, 117, 127, 161, 201, 261	2,3-Me_2_-Glcp	10.50	→ 4,6)-Glcp-(1 →	Zhang et al. (36)

#### NMR Spectrum of PSP-1

**Figure 3:**
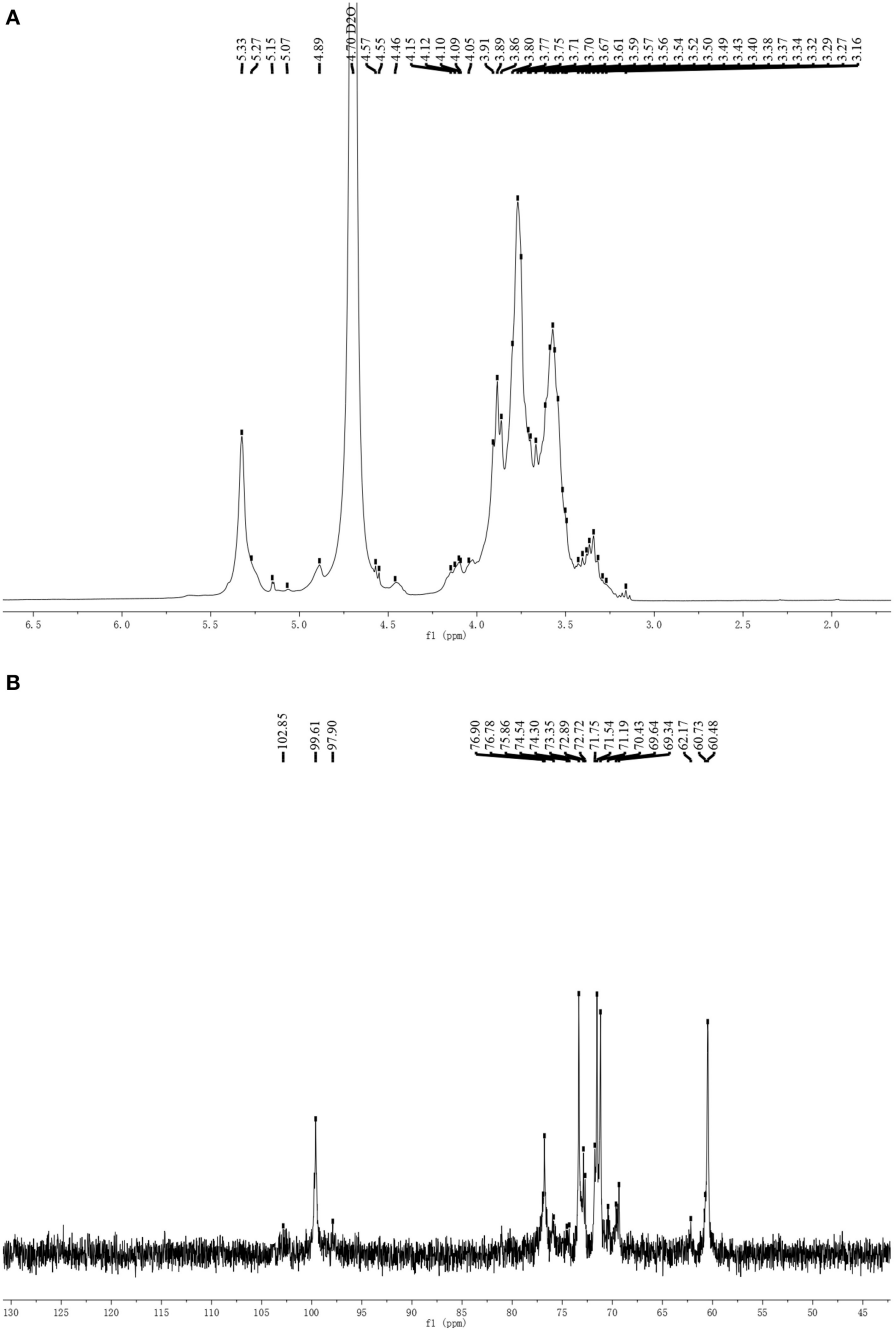
The ^1^H-NMR spectrum **(A)** and ^13^C-NMR spectrum **(B)** of PSP-1.

The ^1^H and ^13^C NMR spectra of PSP-1 are shown in Figures 3A,B. The entire assignment shifts of ^1^H and ^13^C NMR for PSP-1 were identified with reference to the previous literatures (35, 37–39) and illustrated in Table 2. The anomeric proton signals (5.33, 4.89, and 4.46 ppm) and the anomeric carbon signals (99.6, 97.9, and 102.9 ppm) corresponded to H-1 and C-1 of → 4)-α-D-Glc*p*-(1 →, → 4,6)-α-D-Glc*p*-(1 →, and β-D-Glc*p*-(1 →. The NMR results were consistent with the results of monosaccharide composition and methylation analysis.

**Table 2:** ^13^C and ^1^ H NMR chemical shifts (ppm, δ) for PSP-1.

**Sample**	**H-1/C-1**	**H-2/C-2**	**H-3/C-3**	**H-4/C-4**	**H-5/C-5**	**H-6/C-6**	**Reference**
→ 4)-α-D-Glcp-(1 →	5.33/99.6	3.56/71.5	3.91/73.3	3.59/76.8	3.78/71.2	3.77/60.5	Ghosh et al. (37)
β-D-Glcp-(1 →	4.46/102.9	3.34/72.9	3.40/75.9	3.37/69.3	3.67/76.9	3.76/60.7	Agrawal (38); Ghosh et al. (37)
→ 4,6)-α-D-Glcp-(1 →	4.89/97.9	3.49/71.2	3.16/71.5	3.67/76.8	3.89/70.4	4.02/69.4	Shi et al. (39)

Combining all the results from monosaccharide composition analysis, the methylation analysis, and the NMR spectroscopy, the structure of PSP-1 was a water-soluble polysaccharide mainly composed of → 4)-α-D-Glcp-(1 →, → 4,6)-α-D-Glcp-(1 → and β-D-Glcp-(1 → groups. Among them, the content of → 4)-α-D-Glcp-(1 → was the highest. These findings suggest that the main chain of the polysaccharide is → 4)-α-D-Glcp-(1 →, while the branch chain was β-D-Glcp-(1 → and linked at the C6-position of → 4,6)-α-D-Glcp-(1 →.

### Antitumor Activity of PSP-1

#### Inhibitory Effect of PSP-1 on HepG2 Cells

**Figure 4:**
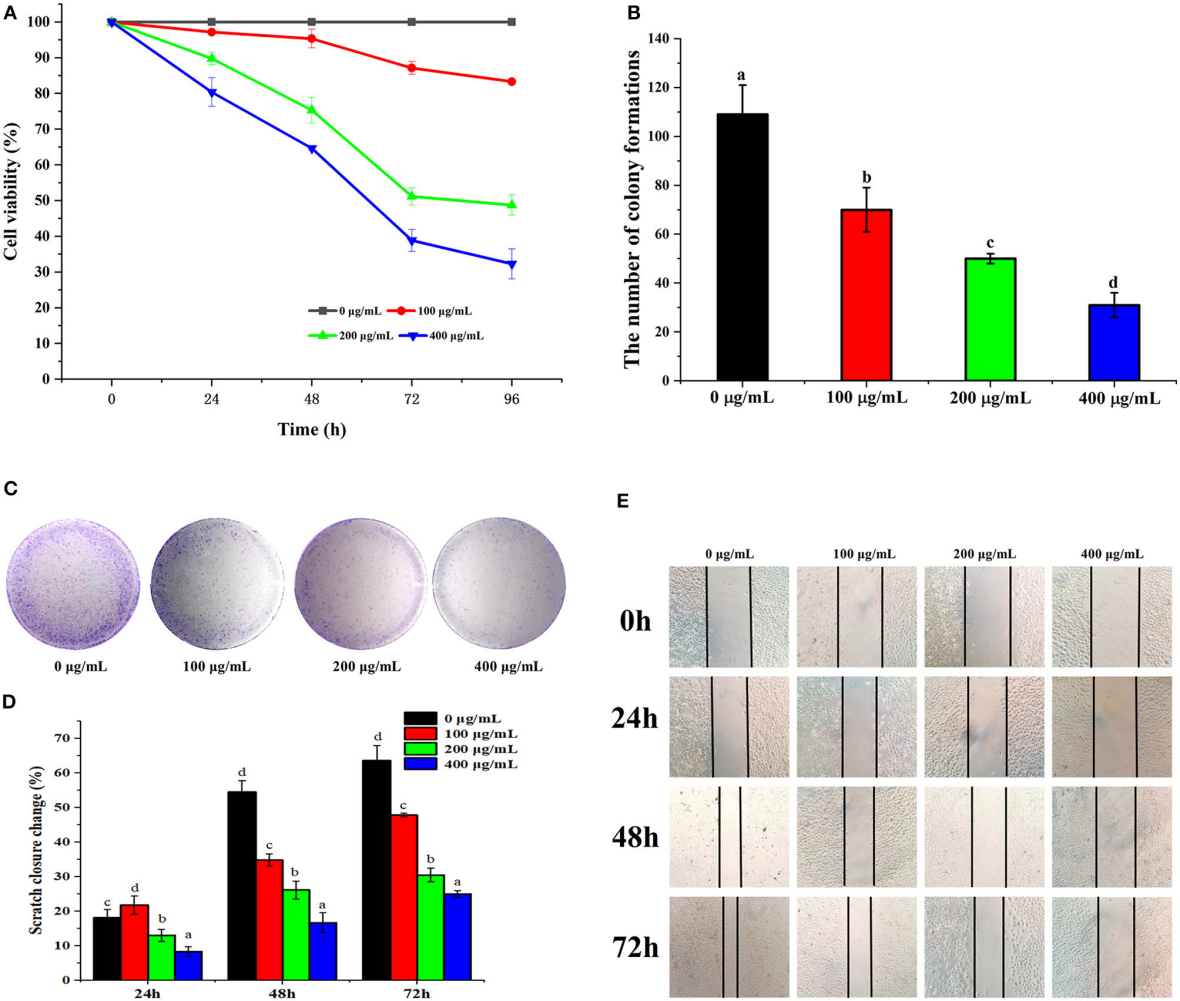
PSP-1 inhibits HepG2 cells proliferation *in vitro*. HepG2 cells were treated with different concentration (0, 100, 200, 400 μg/mL) of PSP-1 for 0, 24, 48, 72, and 96 h. The effect of PSP-1 on cell proliferation was evaluated by MTT assay. **(A)** The clonogenicity of the indicated cells were detected after treatment with different concentration of PSP-1. **(B,C)** Scratch image and scratch closure change of HepG2 cells after treatment with different concentration of PSP-1 **(D,E)**. Data are presented as the mean ± SD, *n* = 3. Different letters above the bars indicate significant difference.

To evaluate the effects of PSP-1 on the proliferation of HepG2 cells, HepG2 cells were treated with PSP-1 or vehicle and analyzed by MTT and colony formation assays. The results of the MTT assay indicated that the proliferation of HepG2 cells treated with PSP-1 was significantly inhibited compared to the control (*P* < 0.05) (Figure 4A). The MTT results further confirmed that HepG2 cells were more susceptible to PSP-1 at a dose of 72 h.

In the colony formation assay, HepG2 cells treated with different concentrations of PSP-1 (100, 200, and 400 μg/mL) formed fewer colonies than the untreated control group, a phenomenon that was concentration-dependent (Figures 4B,C). The results indicate the inhibitory effect of PSP-1 on the clonogenic ability of HepG2 cells.

#### Effect of PSP-1 on Cell Migration

Wound healing assays are commonly used to evaluate tumor cell invasiveness and migration. As shown in Figure 4D, PSP-1 was found to inhibit the wound healing of HepG2 cells in a time-dependent manner. The scratch closure change in Figure 4E indicates that PSP-1 can significantly prevent wound healing. At a PSP-1 concentration of 0, 100, 200, and 400 μg/mL, after 72 h, the rate of scratch closure change of PSP-1 was 63.64, 47.83, 30.43, and 25.00%, respectively. These results indicate that PSP-1 is able to inhibit the migration of HepG2 cells.

#### Effect of PSP-1 on Cell Morphology

**Figure 5:**
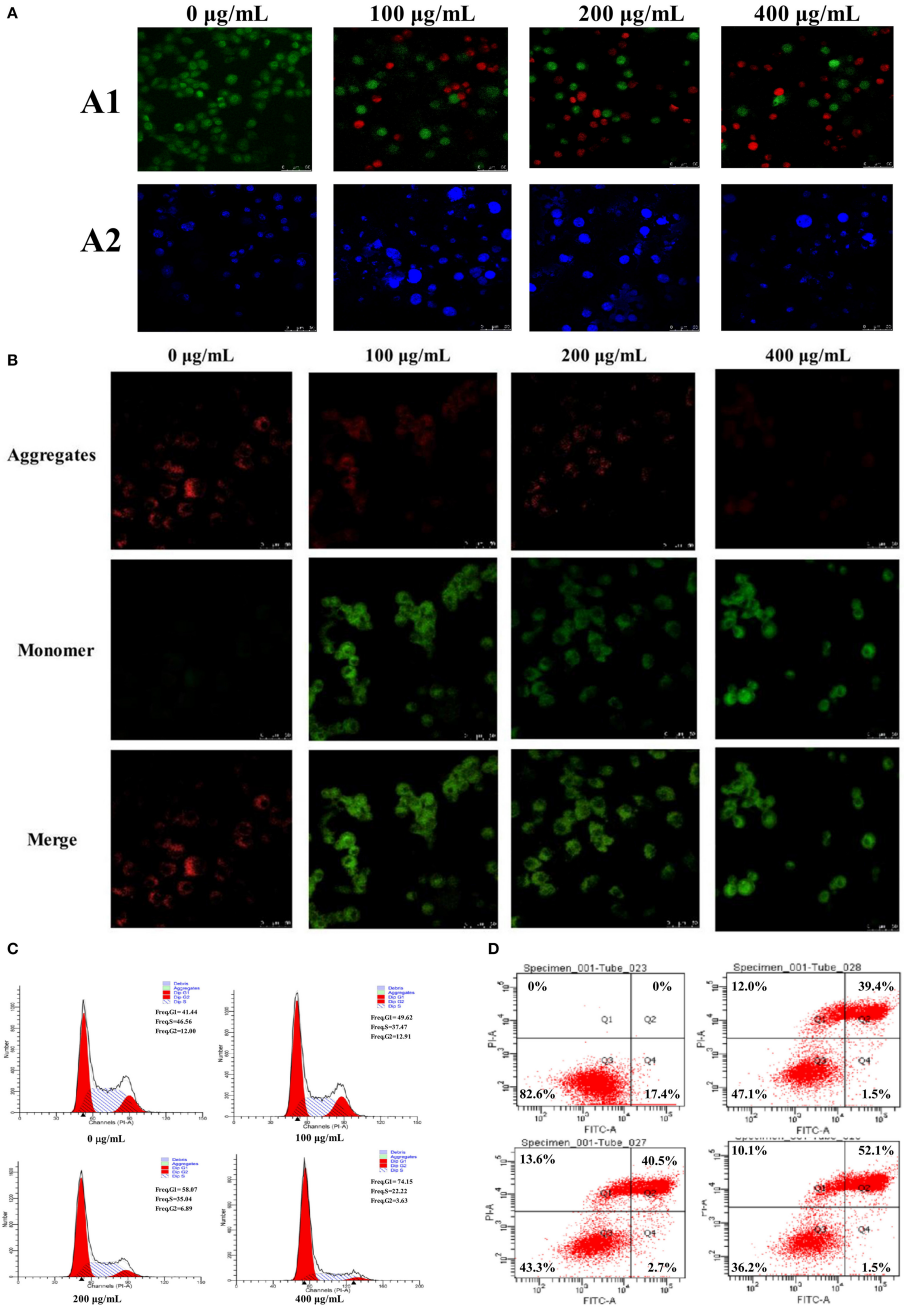
The morphological changes of HepG2 cells treated with 0, 100, 200, and 400 μg/mL of PSP-1 for 72 h **(A)**, the morphological characterization of HepG2 cells was observed and photographed under a Confocal laser scanning microscope **(A1)**, nuclear morphological changes induced by PSP-1 in HepG2 cells after DAPI staining. **(A2)** Effect of PSP-1 on MMP **(B)** Cell cycle progression was assessed using propidium iodide staining detected by fluorescence activated cell sorting. **(C)** The apoptotic rates of the indicated cells induced by PSP-1 at different concentrations for 72 h were detected by ANNexin V/PI double-staining asssy **(D)**.

The apoptosis of HepG2 cells after treatment with PSP-1 was observed using LSCM (Figure 5A1). There was almost no apoptosis of HepG2 cells in the control group under LSCM. Compared to the HepG2 cells in the control group, the number of living HepG2 cells decreased and the number of apoptotic cells increased significantly after 72 h of incubation with an increasing PSP-1 concentration. The number of apoptotic cells was the highest at a concentration of PSP-1 of 400 μg/mL. These results indicated that SPS-1 exerted a significant inhibitory effect on the growth and proliferation of HepG2 cells, with an apoptosis effect that was concentration-dependent.

Hochest 33342 staining was used to observe the PSP-1-induced apoptotic nucleus of HepG2 cells. After Hoechst 33342 staining was performed on HepG2 cells incubated with different concentrations of PSP-1 for 72 h, the nuclei morphology of HepG2 cells were detected using LSCM. The results showed that HepG2 cells treated with PSP-1 exhibited nuclear apoptotic morphologies, such as condensed chromatin, nuclear pyknosis, and apoptotic bodies (Figure 5A2), but no evidence of abnormal nuclear morphology was present in the control group, which displayed intact nuclei. These results suggested that PSP-1 might induce HepG2 cell apoptosis by damaging its nucleus.

#### Determination of Mitochondrial Membrane Potential (MMP, Δψm)

Tetrechloro-tetraethylbenzimidazol carbocyanine iodide (JC-1) is a fluorescent probe that is widely used to detect the mitochondrial membrane potential (MMP, Δψm). When MMP is high, JC-1 aggregates in the matrix of mitochondria to form J-aggregates, which produce red fluorescence. When the mitochondrial membrane potential is low, JC-1 does not gather in the matrix of mitochondria and exists in monomer form, which generates green fluorescence. Therefore, changes in mitochondrial membrane potential were detected by changes in fluorescence color. The decrease in mitochondrial membrane potential is a landmark event in the early stages of apoptosis. Changes in the membrane potential of HepG2 cells treated with different concentrations of PSP-1 for 72 h are shown in Figure 5B. In the absence of PSP-1, HepG2 cells had intact membranes and exhibited uniform red fluorescence. However, as the concentration of PSP-1 increased, a decrease in red fluorescence and an increase in green fluorescence were observed, indicating that the mitochondrial membrane potential of HepG2 cells decreased significantly in a concentration-dependent manner after PSP-1 treatment. When J-aggregates and monomers merged, the cells emitted yellow-green fluorescence, indicating cell apoptosis and even necrosis. Taken together, the results of merged staining indicated that PSP-1 induces apoptosis in cancer cells.

#### Effect of PSP-1 on Cell Cycle and Apoptosis

Cell cycle analysis showed that the cell percentage markedly increased in the G1 phase and declined in the S phase after 72 h of PSP-1 treatment compared with the vehicle group (Figure 5C). At a PSP-1 concentration of 400 μg/mL, PSP-1 significantly increased the portion of HepG2 cells in G1 phase from 41.44 to 74.15% and significantly decreased the portion of HepG2 cells in S phase from 46.56 to 22.22% for HepG2 cells. These results suggested that PSP-1 could induce HepG2 cell cycle arrest in G1 phase.

Next, we measured the effect of PSP-1 on apoptosis in HepG2 cells. The cells were treated with different concentrations of PSP-1 for 72 h, and the apoptosis rate was analyzed by flow cytometry. The results showed that the rate of apoptosis in HepG2 cells was significantly higher in the PSP-1 group than in the vehicle group in a concentration-dependent manner (Figure 5D). At a PSP-1 concentration of 400 μg/mL, the cell population of total apoptotic cells increased from 17.4 to 53.6% in HepG2 cells. These results indicate that PSP-1 significantly inhibited proliferation and induced the apoptosis of HepG2 cells in *vitro*.

#### PSP-1 Activated Caspase-3 and-9

A molecular hallmark of apoptosis is the activation of caspases, which are a family of intracellular aspartate-specific cysteine proteases that execute cell death through proteolytic cleavage in the induction of apoptosis (40). To determine whether caspase activation contributes to PSP-1-induced apoptosis, the activities of initiator caspase (caspase-9) and effector caspase (caspase-3) were assessed using colorimetric assay kits after the treatment of HepG2 cells with PSP-1 (0, 100, 200, and 400 μg/mL) for 72 h. The results showed a concentration-dependent increase in the activities of caspase-9 and−3 in the PSP-1-treated cells (Table 3), suggesting the simultaneous involvement of the intrinsic apoptotic pathways in PSP-1-induced apoptosis in HepG2 cells. The polysaccharides from *Artemisia annua* L. (Huang Huahao) also showed a similar mechanism of inducing apoptosis in HepG2 cells (40).

**Table 3:** Caspase-3 and caspase-9 activity of HepG2 cell after PSP-1 treatment.

**Sample**	**Caspase-3 (×10^**6**^ U/mg prot)**	**Caspase-9 (×10^**6**^ U/mg prot)**
Control	8.92 ± 0.86^a^	8.17 ± 0.83^a^
100 μg/mL	10.87 ± 0.96^b^	10.54 ± 0.72^b^
200 μg/mL	12.62 ± 0.36^bc^	12.46 ± 0.75^c^
400 μg/mL	14.22 ± 1.46^c^	13.57 ± 0.29^c^

*Data were shown as means ± S.D (n = 3). Numbers followed by different letters are significantly different at the level of p < 0.05 according to the Duncan test*.

The antitumor activity of polysaccharides is strongly attributed to their structural features, including their microstructure, monosaccharide composition, Mw, glycosidic linkages, and triple-helix conformation (25, 27, 41). Previous studies have reported that polysaccharides with a smooth lamellar structure exhibit high levels of antitumor activity, which made the active site of action of polysaccharides fully exposed and contributed to its anti-tumor activity (25). Moreover, polysaccharides with lower molecular weights have been found to exhibit stronger binding to receptors on the surface of immune cells due to the occurrence of cross-link receptors, which show higher tumor inhibition due to stronger immune responses (41, 42). The polysaccharide with → 4)-α-D-Glcp-(1 → structure has high levels of immunoregulatory activity (43), and the rank of glycogen is closely related to its biological activity. Highly branched glycogen is more conducive to antitumor and immunomodulatory activities (44). In this study, the low Mw and the smooth lamellar structure in PSP-1 may be the main factors contributing to its antitumor activity.

## Conclusions

In this study, a water-soluble polysaccharide (PSP-1) was obtained from *Polygonatum sibiricum*. The molecular weight of PSP-1 was approximately 38.65 kDa with a particle size of approximately 101.3 nm. The results of monosaccharide analysis, methylation analysis, and NMR indicated that PSP-1 is mainly composed of → 4)-α-D-Glcp-(1 →, → 4,6)-α-D-Glcp-(1 → and β-D-Glcp-(1 → groups, which has a backbone consisting of a → 4)-α-D-Glcp-(1 → backbone with the substitution at O-6 with the β-D-Glcp-(1 → residues. PSP-1 was found to exert potential anti-hepatocellular activity *in vitro*, which could cause nuclear damage and decrease the mitochondrial membrane potential of HepG2 cells. Moreover, PSP-1 was found to significantly inhibit the proliferation and induce the apoptosis of HepG2 cells *in vitro*, increasing the activity of caspase-9 and -3 in the intrinsic apoptotic pathways to induce apoptosis in HepG2 cells. Taken together, our results clearly indicate that PSP-1 shows potential as an antitumor agent. Meanwhile, further research is needed to explore more antitumor mechanisms *in vitro* and *vivo*.

## Data Availability Statement

The original contributions presented in the study are included in the article/Supplementary Material, further inquiries can be directed to the corresponding author/s.

## Author Contributions

ML and YuL: investigation, data curation, methodology, formal analysis, writing—original draft, and software. HZ, YaL, WW, and SY: methodology, formal analysis, and writing—review and editing. XH and MS: data curation, validation, software, investigation, data curation, software, and investigation. RW: supervision, funding acquisition, and project administration. JW: conceptualization, supervision, project administration, writing review and editing.

## Funding

This work was supported by the Joint project of National Natural Science Foundation of China [Grant No. U20A20400]. The Liaoning Provincial Natural Science Foundation regional joint fund project [2020-MZLH-34]. Shenyang young and middle-aged science and technology innovation Leading Talents Project [RC200495]. Guiding Plan of Natural Science Foundation of Liaoning Province [2019-ZD-0714] is revised to Shenyang Science and technology innovation platform project [21-103-0-14, 21-104-0-28].

## Conflict of Interest

The authors declare tha the research was conducted in the absence of any commercial or financial relationships that could be construed as a potential conflict of interest.

## Publisher's Note

All claims expressed in this article are solely those of the authors and do not necessarily represent those of their affiliated organizations, or those of the publisher, the editors and the reviewers. Any product that may be evaluated in this article, or claim that may be made by its manufacturer, is not guaranteed or endorsed by the publisher.
