# Corrigendum: Anti-cancer potential of polysaccharide extracted from *Polygonatum sibiricum* on HepG2 cells *via* cell cycle arrest and apoptosis

**DOI:** 10.3389/fnut.2022.1054565

**Published:** 2022-10-13

**Authors:** Mo Li, Yumeng Liu, Henan Zhang, Yanfeng Liu, Weiming Wang, Shengbo You, Xinyu Hu, Meijun Song, Rina Wu, Junrui Wu

**Affiliations:** ^1^College of Food Science, Shenyang Agricultural University, Liaoning Engineering Research Center of Food Fermentation Technology, Shenyang Key Laboratory of Microbial Fermentation Technology Innovation, Shenyang, China; ^2^College of Criminal Science and Technology, Criminal Investigation Police University of China, Shenyang, China; ^3^Heilongjiang Academy of Traditional Chinese Medicine, Harbin, China; ^4^Institute of Crop Germplasm Resources, Shandong Academy of Agricultural Sciences, Jinan, China

**Keywords:** *Polygonatum sibiricum* polysaccharide, structural characterization, antitumor activity, HepG2 cells, cell apoptosis

In the published article, there was an error in Figure 5B. The effect of PSP-1 (200 μg/mL) on MMP in Figure 5B was wrongly used. The corrected Figure 5B and its caption appear below.

**Figure 5:**
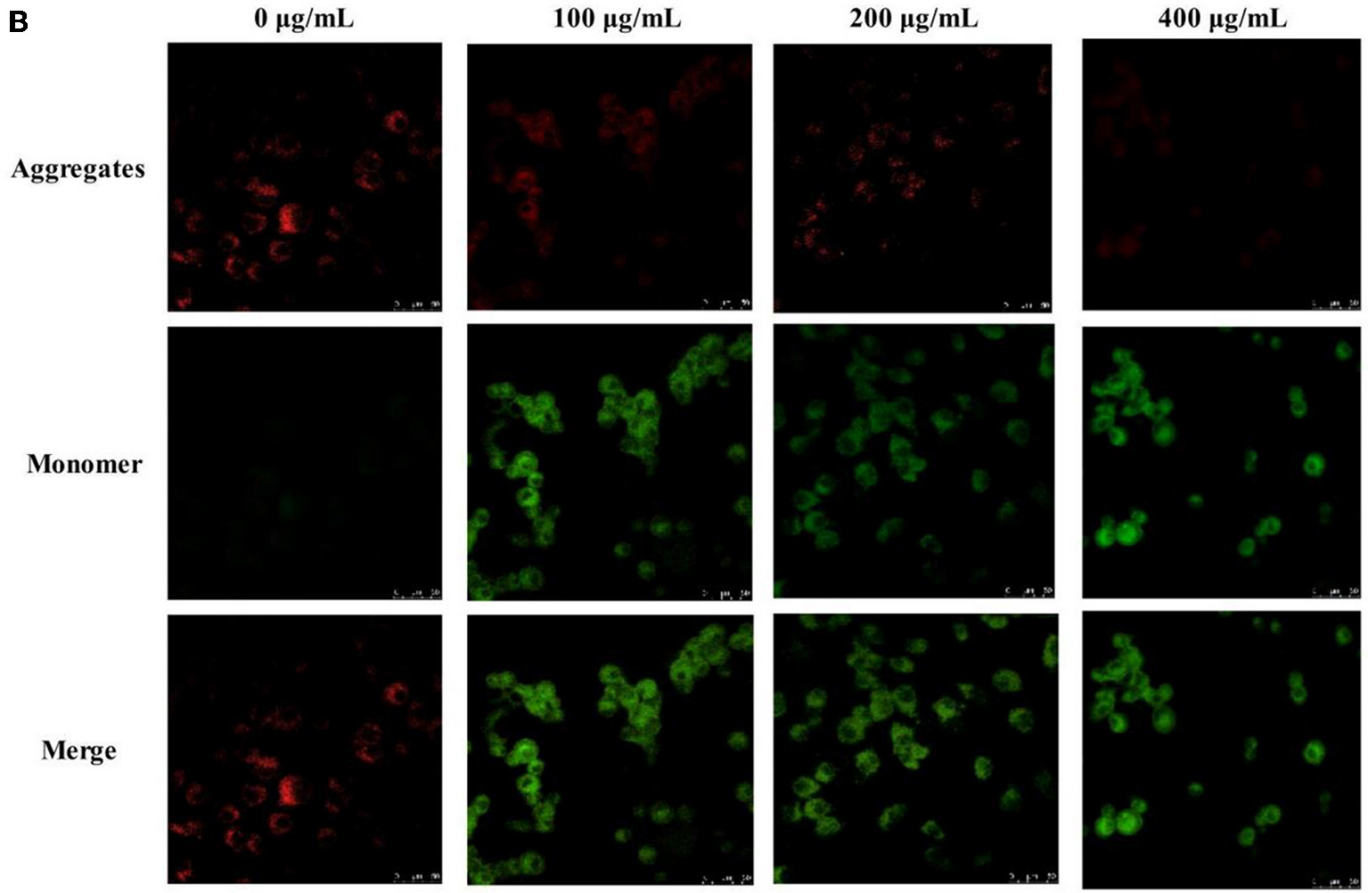
The morphological changes of HepG2 cells treated with 0, 100, 200, and 400 μg/mL of PSP-1 for 72 h **(A)**, the morphological characterization of HepG2 cells was observed and photographed under a Confocal laser scanning microscope **(A1)**, nuclear morphological changes induced by PSP-1 in HepG2 cells after DAPI staining. **(A2)** Effect of PSP-1 on MMP **(B)** Cell cycle progression was assessed using propidium iodide staining detected by fluorescence activated cell sorting. **(C)** The apoptotic rates of the indicated cells induced by PSP-1 at different concentrations for 72 h were detected by ANNexin V/PI double-staining asssy **(D)**.

The authors apologize for this error and state that this does not change the scientific conclusions of the article in any way. The original article has been updated.

## Publisher's note

All claims expressed in this article are solely those of the authors and do not necessarily represent those of their affiliated organizations, or those of the publisher, the editors and the reviewers. Any product that may be evaluated in this article, or claim that may be made by its manufacturer, is not guaranteed or endorsed by the publisher.

