# Supplementary material for: Anti-cancer Potential of Polysaccharide Extracted From Polygonatum sibiricum on HepG2 Cells via Cell Cycle Arrest and Apoptosis
Source: Front Nutr. 2022 Jul 4;9:938290. doi: 10.3389/fnut.2022.938290 (PMC9320318; doi:10.3389/fnut.2022.938290)
Supplement: Supplementary file 1 [file Data_Sheet_1.doc]

Supplementary methods:

Supplementary method S1:

Monosaccharide composition analysis

Briefly, PSP-1 (10mg) was dissolved in 5 mL of trifluoroacetic acid (TFA, 2 M) in a sealed tube and hydrolyzed at 110°C for 8 h. Methanol was added to the hydrolysate and evaporated to dryness using a rotary evaporator to remove TFA. The operation was repeated three times. Then, 500 μL of distilled water was added to dissolve the mixture. Next, 100 μL of hydrolysate was mixed with 100 μL of NaOH solution (0.6 M) and 100 μL 1-phenyl-3-methyl-5-pyrazolone (PMP)-methanol solution (0.5 M) and reacted at 70°C for 100 min. After cooling to room temperature, the mixture was neutralized with 200 μL of HCl solution (0.3 M) before dissolving with 1.4 mL of distilled water. After performing extraction three times with 2 mL of chloroform to remove the PMP, the samples were analyzed by HPLC (Agilent 1100; Agilent, Santa Clara, CA, USA). The sample was separated using an Eclipse Plus C18 column (4.6 × 250 mm, 5 μm) and the column temperature was maintained at 30°C. The mobile phase comprised of 0.1 M phosphate buffer (pH 6.7) and acetonitrile (87:13, v/v) at a flow rate of 1.0 mL/min. The UV detection wavelength was 245 nm.

Supplementary method S2:

Methylation analysis

Briefly, PSP-1 (5 mg) was dissolved in 5 mL of anhydrous dimethyl sulfoxide (DMSO), before NaOH (100 mg) was added. After the mixture was stirred for 4 h, methyl iodide (1.5 mL) was added, and the mixture was stored for 2 h at 25°C in the dark and distilled water (4 mL) was added. The products were extracted three times with chloroform (3 mL), repeating until methylation was complete.

The methylated PSP-1 was hydrolyzed with 10 mL trifluoroacetic acid (TFA) (2 mol/L) at 100°C for 8 h. Then, the hydrolysates were dissolved in 4 mL of 1% (w/w) NaOH, and 10 mg NaBH4 was added to reduce the hemiacetal bond, to which 100 μL of glacial acetic acid was added after the reduction. The sample was dried under reduced pressure, followed by acetylation with acetic anhydride (1.5 mL) and pyridine (1.5 mL) at 100°C for 2 h. The final reaction products were extracted with 4 mL of chloroform and analyzed by GC-MS (Agilent 6890-5973 N; Agilent) with an Agilent HP-5MS capillary column. The temperature program was increased from 140°C to 200°C at 10°C/min, held for 5 min, and then increased to 240°C at 8°C/min. The injection temperature was 250°C, the split ratio was 50:1, and the injection volume was 5 μL.

Supplementary method S3:

Cell proliferation assay and colony formation assay

Briefly, HepG2 cells were seeded in 96-well plates at a density of 8 × 10^4^ cells/well and treated with different concentrations of PSP-1 (0, 100, 200, and 400 μg/mL), or fresh cell medium as a control, for 0, 24, 48, 72, and 96 h, respectively. Then, MTT soluble in PBS (0.5 mg/mL, 20 μL) was added into each well and incubated for 4 h. The media was drawn out, and purple-colored crystals were dissolved using DMSO. Absorbance at 490 nm was measured using a microplate reader. Each experiment was repeated in triplicate. The cell viability rate was calculated using the following formula:

$\text{Cell viability rate (\%) }=\frac{A_{sample}}{A_{control}}\times100\%$ (1)

HepG2 cells were seeded in 6-well plates (800 cells/well) and cultured for 14 days until visible colonies were formed. The colonies were fixed and stained with crystal violet. After staining, the plates were washed and air-dried, and the colony numbers were counted. Each experiment was repeated in triplicate.

Supplementary method S4:

Cell migration assay

HepG2 cells were seeded in 6-well microplates at a density of 5 × 10^5^ cells/well and incubated at 37°C for 48 h. Confluent monolayers were wounded with 200 μL tips, giving rise to an acellular 1 mm-wide lane per well. After washing with PBS to remove cell debris, the cells were cultured in complete medium with or without PSP-1 (200 μg/mL). Wounded areas were photographed at 0, 24, 48, and 72 h of incubation in the dark. Image J software was used to analyze the cell migration distance. The migration inhibition rate was expressed as the percentage of scratch closure change according to the following formula:

$\text{Scratch closure change (\%) }=\left[\left(A_{t0}\text{-}A_{tc}\right)/A_{t0}\right]\times100\%$ (2)

where *A_t0_* is the scratch area at time 0 and *A_tc_* is the corresponding scratch area at 24, 48, and 72 h.

Supplementary method S5:

Cell cycle and apoptosis assay

HepG2 cells were plated (2 × 10^5^ cells/well) into 6-well plates and subjected to the indicated treatments. Approximately 1 × 10^6^ cells were detected using a Cell Cycle Analysis Kit (C1052; Beyotime Biotechnology). A flow cytometer was used to detect cell cycle distribution at 488 nm. The cellular DNA content and light scattering analyses were performed using FlowJo 7.6 software.

Annexin V-FITC Apoptosis Detection Kit (C1062M; Beyotime Biotechnology) was used to detect cell apoptosis. Briefly, HepG2 cells were plated (2 × 10^5^ cells/well) in 6-well plates and treated with PSP-1 solution (0, 100, 200, 400 μg/mL) for 72 h. The cells were suspended in 195 μL of Annexin V-FITC binding fluid, 5 μL of Annexin V-FITC, and 10 μL of PI. Following incubation for 20-25 min at room temperature in the dark, another 400 μL of PBS was added and the percentage of apoptotic cells was determined by flow cytometry.
